# Locoregional Lymphatic Delivery Systems Using Nanoparticles and Hydrogels for Anticancer Immunotherapy

**DOI:** 10.3390/pharmaceutics14122752

**Published:** 2022-12-08

**Authors:** Kyeong Jin Cho, Young-Eun Cho, Jihoon Kim

**Affiliations:** 1 Division of Biological Science and Technology, Yonsei University, Wonju 26493, Republic of Korea; 2 Department of Food and Nutrition, Andong National University, Andong 36729, Republic of Korea

**Keywords:** cancer immunotherapy, lymph nodes, drug delivery, nanoparticles, hydrogels

## Abstract

The lymphatic system has gained significant interest as a target tissue to control cancer progress, which highlights its central role in adaptive immune response. Numerous mechanistic studies have revealed the benefits of nano-sized materials in the transport of various cargos to lymph nodes, overcoming barriers associated with lymphatic physiology. The potential of sustained drug delivery systems in improving the therapeutic index of various immune modulating agents is also being actively discussed. Herein, we aim to discuss design rationales and principles of locoregional lymphatic drug delivery systems for invigorating adaptive immune response for efficient antitumor immunotherapy and provide examples of various advanced nanoparticle- and hydrogel-based formulations.

## 1. Introduction

Immunotherapy has garnered attention in anticancer therapy owing to advances in biotechnology and expanded understanding of the complex immune networks in cancer progression [1]. Cancer cells create immunosuppressive “cold” tumor microenvironments to evade the body’s immune surveillance [2]. Immune checkpoint blockades (ICBs), such as antagonistic cytotoxic T-lymphocyte-associated protein (CTLA)-4, anti-programmed cell death (PD)-1, and anti-PD-ligand (PD-L)-1 monoclonal antibodies (mAbs), which are clinically approved by the U.S. Food and Drug Administration (FDA), are representative immunotherapeutics that reprogram these “cold” tumors to “hot” tumors that are not only characterized by abundant cytotoxic CD8^+^ T cells (CTLs) and natural killer (NK) cells, but are also responsive to these immune cells [2,3]. The therapeutic effects of chemotherapy, the most widely utilized anticancer methodology in the clinic, are found to be either mainly or partially dependent on direct or indirect immuno-modulatory effects of the drugs. As a result, recent re-evaluation and re-purposing of the traditional chemotherapeutics in terms of chemoimmunotherapy have accelerated [4,5]. Accordingly, the anticancer therapy paradigm has been shifting from being heavily focused on cancer cells to a comprehensive consideration of localized tumor microenvironment and systemic immune responses [1,2,4]. 

Current anticancer immunotherapy can be roughly categorized into two strategies: Initiation of anticancer immunity by improving the susceptibility of cancer cells to immune cells to exert anticancer effects in tumor microenvironments. This strategy includes the induction of immunogenic cell death (ICD) to release adenosine triphosphate (ATP), calreticulin (CRT), and high mobility group box protein 1 (HMGB-1) for the recruitment of dendritic cells (DCs) that phagocytize cancer cells or their antigens [4,6,7,8,9], suppression of immunosuppressive pathways of cancer cells [10] or cancer-associated ECM-resident cells [11], or inhibition of the signals associated with the immune checkpoints between cancer cells and immune cells [3,7,12,13,14]. Accordingly, nano-sized materials that can efficiently deliver drugs to tumor tissues via enhanced permeation and retention (EPR) serve as one of the attractive tools to achieve efficient anticancer immunotherapy [4,15,16,17]. 

The lymph nodes (LNs)-centered anticancer immunotherapy to achieve efficient expansion of immune cells in the secondary lymphoid tissues, followed by increased infiltration in tumor microenvironments serves as the second strategy [1,5,18,19,20,21,22,23,24,25,26,27,28,29,30]. LNs are the primary tissues for adaptive immunity. In fact, LNs instruct and expand antigen-specific CTLs via antigen-presenting cells (APCs), such as DCs [1,18,19,20,21,22]. The importance of LNs in adaptive anticancer immunotherapy has been emphasized recently. Francis et al. reported that intradermal (i.d.) injections of ICBs to the tissue ipsilateral (i.l.) to the tumor allows their preferential accumulation in draining LNs (dLNs) over tumor tissues, leading to similar therapeutic effects to intratumoral (i.t.) administration enabling tumor and tumor dLN (TdLN) accumulation [31]. Similarly, Manspeaker et al. demonstrated that locoregional i.l. administration for TdLNs targeting led to a higher anticancer therapeutic index than intravenous (i.v.) administration, mainly targeting the tumor microenvironment in the combination therapy with the PD-1 monoclonal antibody (aPD-1 mAb) and nanoparticles (NPs) loaded with paclitaxel (PTX) [5]. Even in the aforementioned immunotherapy strategy that primarily targets the tumor microenvironments, DCs migrated from peripheral tissues to the LNs after the uptake of antigens produced via natural apoptosis or by cytotoxic drugs, initiate and propagate the anticancer immunotherapy.

The pharmacokinetics of immuno-modulatory drugs govern therapeutic effects, side effects, and patient compliance regarding cancer immunotherapy [16,32,33,34,35,36,37,38]. A hydrogel (HG) is a 3D polymeric network that contains water and serves as one of the important classes of drug delivery systems for the sustained release of drugs. Indeed, i.t. administration of HGs has been reported to lead to prolonged accumulation of immuno-stimulatory drugs to the tumor microenvironment, leading to a higher anticancer therapeutic index than that obtained with bolus delivery [32,33,34]. In addition, locoregional i.l. administration of ICBs-loaded HGs resulted in prolonged and higher accumulations of mAbs in TdLNs, followed by improved anticancer immunotherapy [31,35].

After considering numerous reviews that enabled an extensive and intensive discussion of the design rationales and examples of biomaterials for the delivery of immuno-therapeutics to the tumor microenvironment [1,4,16,17,36,37,38], we aimed to discuss the principles, design rationales, and recent progress of lymphatic drug delivery systems to achieve efficient adaptive anticancer immunotherapy. First, the design rationales and examples of advanced NPs required to overcome physiological barriers of lymphatic systems were summarized in conjunction with detailed immune response. Second, several advanced HGs that enable prolonged adaptive immune response in LNs were discussed. Finally, hybrid HGs and NPs systems that allow sustained delivery of drug-loaded NPs to the LNs for efficient anticancer immunotherapy were introduced. 

## 2. Advanced NPs for LNs Targeting and Adaptive Cancer Immunotherapy

### 2.1. Basic Criteria for the Lymphatic Delivery of NPs and Immunomodulatory Drugs

LNs-centered adaptive cancer immunotherapy requires a cascade pathway. First, NPs containing immuno-modulatory drugs should drain into the LNs [18,19,21]. In locoregional administration of NPs, such as i.d., i.t., subcutaneous (s.c), or intramuscular (i.m.), the transport of NPs to dLNs depends on interstitial flow, a bulk fluid flow for transit to the initial lymphatics within tissues [18,39]. NPs with a hydrodynamic size ranging from 10 to 50 nm can efficiently pass through the pores of the extracellular matrix of tissues in bulk [40,41,42,43,44,45]. However, the size threshold for delivery into the lymphatics can differ based on the flexibility and rigidity of the nanomaterials [40,41,42,43,44,45,46]. Further, NPs with negative surface charges display a higher transport to the lymphatics than NPs with positive charges, which is due to the negative charge of the ECM [43,47,48,49,50]. In this regard, the surface modification of polyethylene glycol (PEG) to facilitate anti-biofouling [51] and neutralization of surface charges can improve the lymphatic delivery efficacy of NPs [52,53,54,55]. In particular, lymph-homing and migratory APCs contribute to the cell-mediated lymphatic delivery of NPs not only via phagocytosis [27,41,42,44,52], but also selective interaction with NPs engineered for modification with mannose, mannan, hyaluronic acid, hyaluronan, and antibodies to bind to the receptors of APCs [56,57,58,59,60,61,62,63,64]. During systemic delivery of NPs, such as intravenous (i.v.) or intraperitoneal (i.p.), NPs in the bloodstream can access LNs via high endothelial venules (HEVs) enabling transmigration of lymphocytes interacting with peripheral node addressin (PANd) in LNs [18,19,65,66,67,68,69], which can be improved when NPs are decorated with PNAd-targeting antibodies [70,71,72]. Second, immuno-modulatory drugs that reach the afferent lymphatic vessels of LNs can access the LN parenchyma when NPs or drugs released from NPs are smaller than 70 kDa in size [73]. APCs that have already encountered NPs and immuno-modulatory drugs, or other migratory immune cells interacting with NPs containing immuno-modulatory drugs, facilitate cell-mediated delivery to the cortex and paracortex regions of LNs, where adaptive immune responses occur [74,75,76,77,78].

### 2.2. Advanced NPs to Exploit Passive Transport to the LNs

Passive transport involves the delivery of cargos to LNs by exploiting interstitial flow toward LNs within the ECM. Considering the physicochemical properties that enable transport through the ECM from peripheral tissues to LNs [18,39,40,41,42,43,44,45,46,47,48,49,50,51], efficient lymphatic delivery of small molecular immuno-modulatory drugs requires advanced drug delivery systems not only to tune the physicochemical properties of cargos comprising drugs and drug delivery systems, but also to allow the loading and release of drugs at LNs. Depending on the intrinsic properties of immuno-modulatory drugs, various strategies can be employed for drug loading, such as hydrophobic/hydrophilic interactions, electrostatic interactions, nucleotides hybridization, protein engineering, or chemical conjugation. Recent advancements in NP design for efficient lymphatic passive transport are discussed below.

#### 2.2.1. Physical Load; Hydrophobic/Hydrophilic Interactions

Micelles are one of the representative drug delivery systems that allow the loading of hydrophobic drugs into the hydrophobic core of structures formed via the self-assembly of block-copolymers comprising hydrophilic and hydrophobic segments in an aqueous solution [79]. Thomas et al. utilized a micelle physically entangled by poly(propylene sulfide) (PPS) to explore the adjuvant potential of paclitaxel (PTX) in LNs. PTX-loaded PPS micelles (~30 nm) administered i.l. caused moderate suppression of B16F10 melanoma tumor growth, and were more efficient than those administered c.l. (i.d. administration to the tissue contralateral to the tumor) [80]. The mechanism underlying the therapeutic effects of PTX is ascribed to the activation (CD86^+^, CD40^+^ and/or MHCII^+^) and expansion of conventional DCs (cDCs, CD45^+^CD11b^+^CD11c^+^) in LNs [5,80,81]. In particular, i.l. or i.t. administration of free aPD-1 mAbs combined with PTX-loaded PPS micelles administered i.l. significantly expanded stem-like CD8^+^ T cells (Tcf-1^+^Tim-3^-^PD-1^+^ CD45^+^CD3^+^CD8^+^) systemically in triple-negative breast cancer (TNBC) E0771 tumor models after selective accumulation in TdLNs, leading to the control of distant and local tumors [5]. This series of projects suggest a strategy to re-purpose traditional drugs by exploiting micelles as lymphatic delivery systems [5,80,81]. 

Contrary to the micelles, lipid NPs and liposomes serve as a carrier for hydrophobic and hydrophilic drugs by incorporating them into the hydrophobic and hydrophilic segments of the self-assembled structures, respectively [23,82]. Hanson et al. utilized lipid NPs to demonstrate the potential of lymphatic accumulation of a stimulator of IFN gene (STING) agonists as a vaccine adjuvant [83]. Indeed, lipid NPs (150 nm) administered s.c. led to increased accumulation of the STING agonists in dLNs and mitigated systemic exposure of the drugs in blood. Compared to soluble STING agonists, the STING agonist-loaded lipid NPs significantly improved the expansion of antigen-specific (tetramer^+^) CD8^+^ T cells (CD45^+^CD3^+^CD8^+^) when co-administered with ovalbumin antigen (OVA), leading to a markedly higher anticancer effect in terms of survival and tumor growth [83]. 

#### 2.2.2. Physical Load; Electrostatic Interactions

Nucleotides and nucleic acids contain phosphate backbones that have negative charges, enabling electrostatic complexation with positive charged-materials for the development of nano-sized particles that have been explored as a non-viral gene delivery option [16,27]. Similarly, nucleotide adjuvants or nucleic acids encoding antigens can be efficiently formulated into NPs via electrostatic complexation for efficient lymphatic delivery. Solid lipid NPs (SLP) containing cationic phospholipids (152 nm) could not only physically load mRNA encoding OVA, tyrosinase-related protein 2 (TRP-2), or point-mutated version of glycoprotein 100 (gp100) that are melanoma self-antigens but also efficiently drain into the dLNs [84], ultimately resulting in improved antigen-specific (tetramer^+^) CD8^+^ T cell (CD45^+^CD3^+^CD8^+^) expansion and efficient tumor regression. Nucleotide-based adjuvants can be delivered to lymphoid tissues using cationic nanomaterials, thereby mitigating systemic non-specific immune response. For example, the s.c. administration of gelatin NPs (272 nm) cationized by quaternary amino groups to electrostatically load CpG oligonucleotides, a Toll-like receptor 9 (TLR9) ligand, led to their selective accumulation in dLNs over the spleen, resulting in the efficient activation of CpG-positive DCs in LNs, suppression of tumor growth, and prevention of splenic follicle destruction [85]. In addition to NPs comprising organic materials, such as lipids and polymers [84,85,86,87], inorganic materials can be utilized to electrostatically load and deliver nucleotides and nucleic acids to the LNs for efficient anticancer immunotherapy. The benefits of inorganic NPs relative to organic NPs include the ease of tuning the size and surface charges and rendering additional functions, such as imaging, photothermal and photodynamic properties, and intrinsic adjuvant effects. For example, triethoxypropylaminosilane-functionalized silica NPs (SiNPs, 30 nm) [88], polyethyleneimine (PEI)-functionalized mesoporous silica NPs (MSN, ~200 nm) [89], and aluminum hydroxide NPs (~85 nm) [90] were explored as CpG lymphatic delivery systems for efficient anticancer immunotherapy; however, their intrinsic adjuvant effects [91] during delivery to the LNs need to be further investigated. The lymphatic transport mechanism of these rigid NPs with large sizes must also be investigated [40,41,42,43,44,45,46]. 

**Figure 1:**
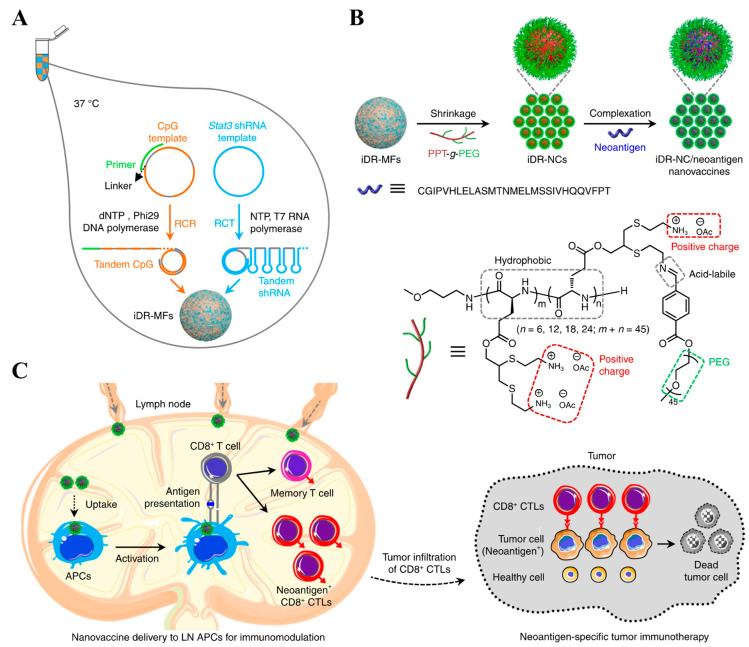
Schematic illustration of the preparation and actions of NPs. (**A**) Microflowers were first synthesized via self-assembly of polymerized DNA and shRNA synthesized using the RCA of DNA templates to encode CpG and *Stat3* shRNA. (**B**,**C**) (**B**) Cationic polymers allowed the shrinking of microflowers to develop NPs capable of (**C**) lymphatic drainage for adaptive immune response. The figure is adapted from [93] with permission from Springer Nature. Copyright 2017 Springer Nature.

In non-viral gene delivery systems, the size of polyplex NPs is inversely proportional to the multivalent electrostatic interactions governed by Coulomb’s Law. Short-length monomeric small interfering RNA (siRNA) resulted in NPs with a size of ~460 nm with linear PEI (LPEI). The formation of a multimerized siRNA through self-crosslinking between siRNA via reducible disulfide bonds could lead to NPs with a size of ~82 nm [92]. The compact structures of NPs in multimerized siRNA are attributed to the higher charge density and more flexible structure than those of monomeric siRNA. Accordingly, polyplex NPs are often developed using multimerized nucleotide-based adjuvants for efficient lymphatic delivery. The Xiaoyuan Chen group utilized rolling circle amplification (RCA) to construct polymerized CpG with additional polymerized shRNA encoding signal transducer and activator of transcription 3 (*Stat3*) to suppress antigen-specific T cell tolerance [93,94]. Microflowers comprising poly(CpG) and poly(sh*Stat3*) can shrink from ~1200 nm to NPs of ~250 nm when complexed with positively charged PPT-*g*-PEG polymer, leading to efficient presentation of antigens on DCs (SIINFEKL^+^ CD45^+^CD11c^+^) in LNs after i.l. administration (Figure 1) [93]. In another study, poly(CpG) microflowers were produced on primers conjugated to the PEG segments of PEG-PLA micelles, enabling the co-encapsulation of a hydrophobic peptide neoantigen to MC38 tumor (herein, adpgk) and a hydrophobic adjuvant (herein, a TLR 7/8 agonist R848) in the core of micelles [94]. The resulting micelle-based microflowers containing poly(CpG), adpgk, and R848 with ~2200 nm size can be condensed into NPs with a size of ~170 nm when complexed with PPT-*g*-PEG. The resulting polyplex micelles were found to have higher LN accumulations than their free formulations following s.c. administration, ultimately leading to a higher expansion of LN-resident DCs (CD45^+^CD11c^+^) and macrophages (CD45^+^F4/80^+^), and systemic antigen-specific (dextramer^+^) CD8^+^ T cells (CD45^+^CD3^+^CD8^+^), and efficient tumor regression [94]. The combined administration of aPD-1 mAbs could also improve the therapeutic index [94].

#### 2.2.3. DNA/RNA Engineering; Spherical Nucleic Acids (SNA) and DNA/RNA Origami

**Figure 2:**
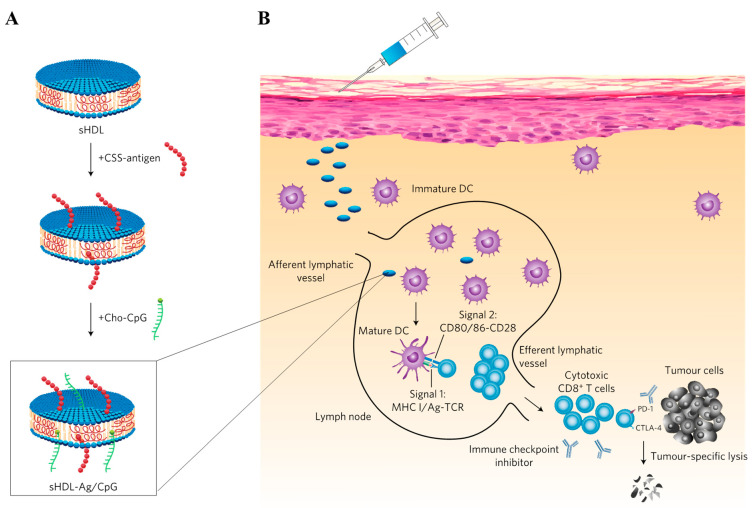
Schematic illustration of the preparation and actions of SNA with liposomal nanodisc cores. (**A**) Antigens were conjugated to liposomal nanodisc via a disulfide bond and cholesterol-CpG were embedded on the phospholipid layers to afford an SNA structure where CpG was exposed to the nanodisc. (**B**) The nanodisc was drained into the LNs to instruct the antigen-specific CD8^+^ T cells in LNs for anticancer immunotherapy. The figure is adapted from [99] with permission from Springer Nature. Copyright 2017 Springer Nature.

Spherical nucleic acids (SNAs), which were first introduced by Chad Mirkin’s group, comprise an NP core and a densely packed nucleic acid layer [95]. SNAs exhibit distinguished properties from bare nucleic acids in terms of cellular uptake, stability against enzymatic degradation, and adsorption with endogenous biomolecules [95,96]. SNA with a 13 nm gold NP (AuNP) core and a CpG shell displayed significant accumulation in the subcapsular sinus of dLNs, but not in the parenchyma [97]. Nevertheless, the CpG-decorated SNA induced the release of higher amounts of interleukin (IL)-12 from dLNs than free CpG, which significantly suppressed tumor growth when combined with an antigenic OVA protein or SIINFEKL peptides [97]. In a study utilizing liposomal SNA (~120 nm) where cholesterol-modified DNA is embedded into the phospholipid membrane of core liposome (80–90 nm), the effects of liposomal membrane fluidity on lymphatic delivery of SNA were examined by varying the lipids with different phase transition temperature (T_c_) [98]. Despite the identical NP size and surface chemistry, a liposomal SNA with higher T_c_ accumulated in a higher quantity in dLNs than that with lower T_c_ at an early time point (2 h after s.c. injection), which led to more efficient suppression of tumor growth [98]. However, the accumulation of liposomal SNA was equivalent at 24 h, regardless of T_c_ [98]. As membrane fluidity is reversely proportional to T_c_, these results imply that a rigid core is beneficial in the rapid lymphatic delivery of SNA, followed by LNs-mediated anticancer immunotherapy. The James Moon groups reported a liposomal nanodisc, in which cholesterol-modified CpG was embedded on the surficial phospholipid membrane and cysteine-functionalized neoantigen peptides were chemically conjugated to the thiol-functionalized phospholipids (Figure 2) [99]. Nanodiscs injected s.c. facilitated higher accumulation of CpG and antigen peptides in the dLNs, enabling efficient antigen-specific (tetramer^+^) CD8^+^ T-mediated anticancer therapy [99]. As numerous kinds of core NPs can facilitate the loading of various hydrophobic and hydrophilic adjuvants and antigens, SNAs with various functions are expected to serve as a platform for lymphatic-mediated anticancer immunotherapy [100,101].

**Figure 3:**
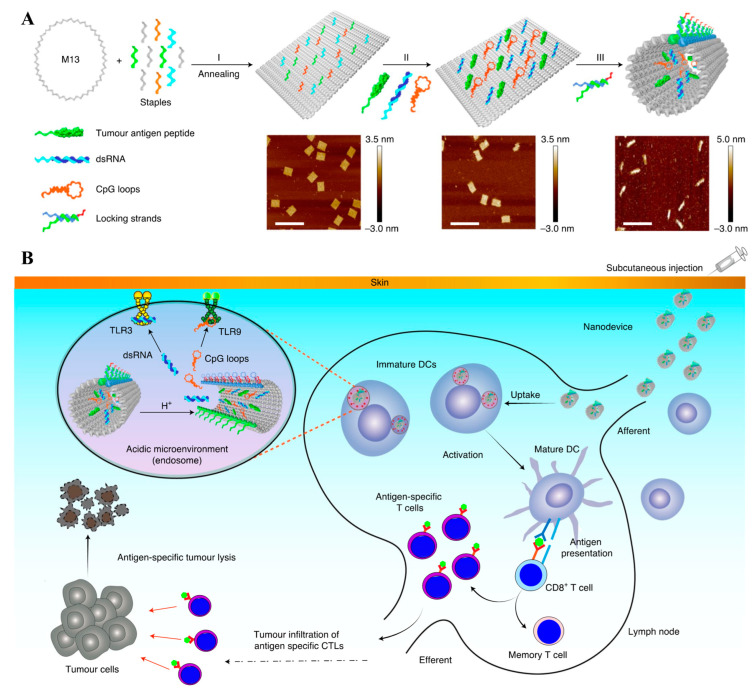
Schematic illustration of the preparation and actions of pH-sensitive DNA nanodevice. (**A**) Rectangle-shaped DNA was prepared using staple strands and M13 bacteriophage DNA, which were further engineered to load tumor peptide-antigen-DNA conjugate antigens and double stranded RNA (dsRNA) adjuvants. pH-sensitive locking strands were then employed to generate tubular DNA nanostructures. (**B**) Mechanism of actions of the DNA nanostructures. pH-sensitive locking DNA was dehybridized to open the tubular structure, exposing the antigens and dsRNA outside to stimulate DC activation and presentation of antigens after draining into the LNs, followed by uptake by DCs, leading to antigen-specific CD8^+^ T cell response for robust anticancer immunotherapy. The figure is adapted from [104] with permission from Springer Nature. Copyright 2021 Springer Nature.

DNA and RNA have been exploited as building blocks for well-defined nanostructures owing to their Watson–Crick base pairing [95,102]. One of the interesting fields include DNA/RNA origamis that fold long single-stranded DNA/RNA scaffolds into an arbitrary structure using short DNA/RNA complementary to the scaffold as a staple [102]. Previously, a DNA tetrahedron structure was demonstrated to display a higher accumulation in dLNs than double stranded linear DNA [103]. The Baoquan Ding groups reported an interesting DNA nanodevice that enabled structural transformation from tubular to sheet under intracellular endosomal pH using DNA origami (Figure 3) [104]. Multiple staple strands were utilized to fabricate a rectangle-shaped DNA origami from M13 bacteriophage DNA, which was further exploited to load tumor peptide antigen-DNA conjugates as an antigen and CpG and double-stranded RNA (dsRNA) as adjuvants via hybridization [104]. pH-responsive DNA was also employed to hybridize the DNA in long edges of rectangle-shaped DNA origami, enabling the formation of a tubular nanostructure with diameter and length of ~19 nm and ~90 nm, respectively, and pH-responsive transformation [104]. Compared to the physical mixture of therapeutics, including peptide antigens, CpG, and dsRNA, the resulting DNA nanodevices could drain into dLNs ~5 times more, eliciting higher expansion and activation (CD86^+^) of DCs (CD45^+^CD11c^+^), followed by enrichment of antigen-specific (tetramer^+^) CTL (IFN-γ+ CD45^+^CD3^+^CD8^+^) [104]. Accordingly, robust antitumor immunotherapy in various tumor models, including MC38, B16F10, and B16F10-OVA, was achieved [104].

#### 2.2.4. Self-Assembled or Recombinant Protein

In addition to their general therapeutic roles, including as antigens or therapeutic antibodies, proteins can serve as a lymphatic drug delivery platform. Owing to size-dependent LN accumulation, self-assembled OVA NPs (~80 nm) were developed by cleaving the disulfide bonds of an OVA antigen protein to change its charges to positive and then complex it with CpG [105]. Coordination among OVA antigen proteins, manganese (Mn^2+^), and meso-2,6-diaminopimelic acid (DAP) was found to facilitate the preparation of nanocomposites with a size of 150 nm [106]. The increased size of these self-assembled antigen NPs led to a significantly higher LN accumulation of antigens than bare antigen proteins [105,106], followed by successful suppression of tumor growth [106]. 

**Figure 4:**
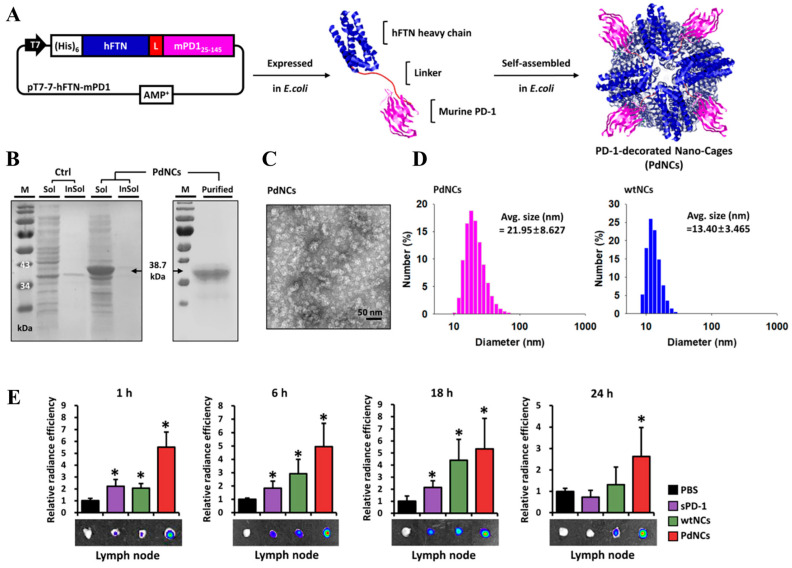
Schematic illustration of the preparation and lymphatic drainage of protein-fusion nanocages (PdNCs). (**A**) Self-assembled PdNCs (**right**) from a fusion protein (**mid**) containing hFTN and aPD-1 were prepared; these PdNCs were synthesized from a plasmid vector (**left**). (**B**) SDS-PAGE of the plasmid vector. (**C**) Representative TEM image of the PdNCs. (**D**) DLS size distribution of PdNCs (**left**) and control nanocages self-assembled from wild hFTN (**right**). (**E**) Quantification of the LN accumulation of PdNCs using ex vivo IVIS. * *p* < 0.05 was analyzed by one-way ANOVA with Tukey’s post-hoc. Figures are reproduced from [108] with permission from Elsevier. Copyright 2021 Elsevier.

Proteins vary in size, 3D structures, and surface characteristics, which critically affect lymphatic delivery. Advances in genetic recombination and protein engineering technology enable not only the preparation of recombinant proteins to present antigens or antibodies but also the tuning of physicochemical properties for self-assembly. Lee et al. demonstrated that human ferritin (hFTN, ~11 nm) exhibited a 3-to-7-fold higher accumulation than *Escherichia coli* DNA binding protein (DPS, 9.5 nm), *Thermoplasma acidophilum* proteasome (PTS, 13.4 nm), and Hepatitis B virus capsid (HBVC, 32.3 nm) [107]. Genetic fusion of RFP and hFTN subunits afforded an engineered hFTN to present a model tumor antigen (herein, red-fluorescence protein (RFP)), which induced conformational changes in proteins. The diameter of the engineered hFTN increased to ~22 nm. The hFTN-RFP protein NPs efficiently drained into the LNs, inducing efficient antitumor immunotherapy against RFP-expressing tumor-bearing models [107]. Owing to the efficient lymphatic transport of hFTN, Kim et al. developed hFTN fusion proteins to present a PD-1 (Figure 4) [108]. The resultant nano-cages had a larger size (~22 nm) than wild-type hFTN (wtNCs, ~13 nm), and they displayed not only a higher affinity against PD-L1 and PD-L2 than soluble PD-1 (sPD-1) but also higher accumulation in dLNs than the monomeric form of sPD-1 and wtNCs. Accordingly, efficient activation (CD86^+^, CD40^+^) of DCs (CD45^+^CD11c^+^) and CD8^+^ T (CD45^+^CD3^+^CD8^+^) cells-mediated inhibition of tumor growth occurred [108]. A further study to examine the mechanism whereby the properties of hFTN facilitate the efficient lymphatic delivery is expected to contribute to the development of advanced lymphatic delivery systems using protein scaffolds.

#### 2.2.5. Chemical Conjugation

Drugs with functional groups that can participate in a chemical reaction can be conjugated to various drug delivery systems, including iron oxide NPs, quantum dots, graphene oxide, liposomes, micelles, etc. [109,110,111,112,113]. Examples include conjugations of CpG and si*Stat3* to linkers on the quantum dots (~150 nm) via a disulfide bond [109], amide conjugations of antigen peptides to lipids to enable hydrophobic interactions with oleic acids on iron oxide NPs (30–45 nm) [110], amide conjugations of antigen peptides to PEG-(poly(maleic anhydride-alt-1-octadecene)) loaded on graphene oxide (20–30 nm) via hydrophobic interactions [111], conjugations of antigen peptides to lipids comprising liposomes (~23 nm) via an amide bond [112], and conjugations of antigen peptides to poly(L-histidine)-poly(ethylene glycol) micelles (~60 nm) via a disulfide bond [113], ultimately leading to a high accumulation of immunomodulatory drugs in the LNs, followed by efficient antitumor immunotherapy. The other examples are discussed in overall manuscript.

### 2.3. Advanced NPs for Active Transport to the LNs

In contrast to passive transport, active transport involves the lymphatic transport of cargos via immune cells. In particular, peripheral DCs encountered immuno-stimulatory agents and antigens can initiate adaptive antitumor immune response in dLNs owing to the intrinsic lymph-homing ability of maturating and activating APCs. A representative strategy involves the construction or functionalization of NPs with mannose & mannan, or hyaluronic acid & hyaluronan with mannose-receptors or CD44, respectively, which are expressed on the surfaces of APCs, such as DCs and macrophages [56,57,58,59,60,61,62,63,64,114,115,116,117]. Ligand-mediated improvement of the lymphatic delivery of cargos was clearly demonstrated with mannose-decorated liposomes (~160 nm) loading poly(I:C) electrostatically [56], tumor cell lysate-loaded chitosan NPs (~120 nm) functionalized with mannose [114], micelles (~95 nm) comprising stearic acid-grafted and mannose-modified chitosan [115], NPs (~310 nm) comprising alginates grafting mannose and OVA proteins [116], mannose-functionalized pH-sensitive micelles (30–50 nm) loading antigens via disulfide bonds [117], and TLR7/8 agonists and OVA proteins loaded poly(lactide-co-glycolide) (PLGA) NPs (~225 nm), whose surface was emulsified with hyaluronic acid [62]. Of note, in these NPs, the size-dependent passive transport mechanisms cannot be omitted from the uptake of drugs and NPs in lymph-homing APCs and APCs-mediated CD8^+^ T cell instructions in LNs. Indeed, by comparing a mannan nanocapsule and a silica NP decorated with mannan, a higher quantity of nanocapsule was found to be accumulated in the dLNs than that of silica NPs, despite similar zeta potential and size (both ~220 nm). These results were ascribed to the high deformability of nanocapsules to pass through a 50–100 nm pore-sized membrane; silica NPs cannot efficiently pass through such membrane [61]. The study findings also implied the partial, but significant contribution of passive lymphatic transport pathways, even in the case of NPs decorated with ligands, to the interaction with lymph-homing APCs.

**Figure 5:**
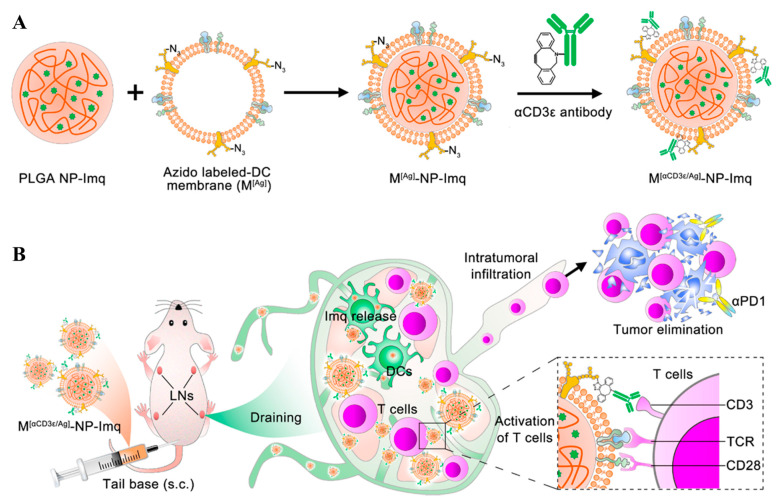
Schematic illustration of the preparation and lymphatic drainage of DC-membrane cloaking NPs. (**A**) Preparation of aCD3 functionalized DC-membrane cloaking NPs by coating imiquimod-loaded PLGA NP with the DC membrane and conjugating aCD3 with the membrane. (**B**) The NPs drained into the LNs and elicited an antitumor immune response after s.c. administration. The figure is adapted from [122] with permission from American Chemical Society. Copyright 2021 American Chemical Society.

The intrinsic biocompatibility of mammalian cell membranes has promoted the development of membrane-cloaked NPs for efficient lymphatic delivery and antitumor immunotherapy [118]. For example, red blood cell (RBC) membranes for the camouflage of PLGA NPs chemically loading antigenic peptide (herein hgp100_25–33_) were modified with mannose (~160 nm) by embedding lipids-PEG-mannose to improve lymphatic active transport of the NPs [119]. Compared to the controls, including PLGA NPs and RBC-cloaked PLGA NPs, the mannose-RBC-cloaked PLGA NPs were more susceptible to uptake by APCs and accumulation into dLNs, and induced more efficient CD8^+^ T (CD45^+^CD3^+^CD8^+^) cell-mediated antitumor effects after i.d. administration [119]. In this regard, cancer cell membranes containing tumor-associated antigens could be exploited as a substitute for biomaterials containing mammalian cell membranes and antigens [120]. In particular, DC membranes containing CCR7, L-selectin, and integrins can be an interesting source for camouflaging NPs to exhibit homologous targeting to LNs [121]. Indeed, functionalization with DC membranes significantly promoted the LN accumulation of OVA protein-loading micelles synthesized using stearic acid- and histidine-grafted chitosan polymers (~150 nm), and improved the activation (CD86^+^ and CD40^+^) of DCs (CD45^+^CD11c^+^) in dLNs and expansion of CD8^+^ T cells (CD45^+^CD3^+^CD8^+^) in tumors [121]. DC membranes can be engineered to present antigens on major histocompatibility complex (MHC) and CD28 co-stimulator before being used for cloaking NPs (Figure 5) [122]. Further functionalization of matured DC membrane-camouflaged NPs (~180 nm) with CD3 antibodies was reported to enable significant regression of tumor growth owing to efficient lymphatic accumulation and retention of NPs via the combined lymph-homing effects of DC membranes and T cell binding effects of the CD3 antibodies [122]. The resultant NPs containing imiquimod led to efficient antitumor effects, with significant activation of DCs (CD45^+^CD11c^+^ CD80^+^CD86^+^) and macrophages (CD80^hi^ CD45^+^CD11b^+^F4/80^+^) and expansion of CD8^+^ T cells (CD45^+^CD3^+^CD8^+^) [122]. Based on these criteria, fused membranes from DCs and cancer cells have been explored in the development of membrane-cloaked NPs for antitumor immunotherapy [123,124]. 

**Figure 6:**
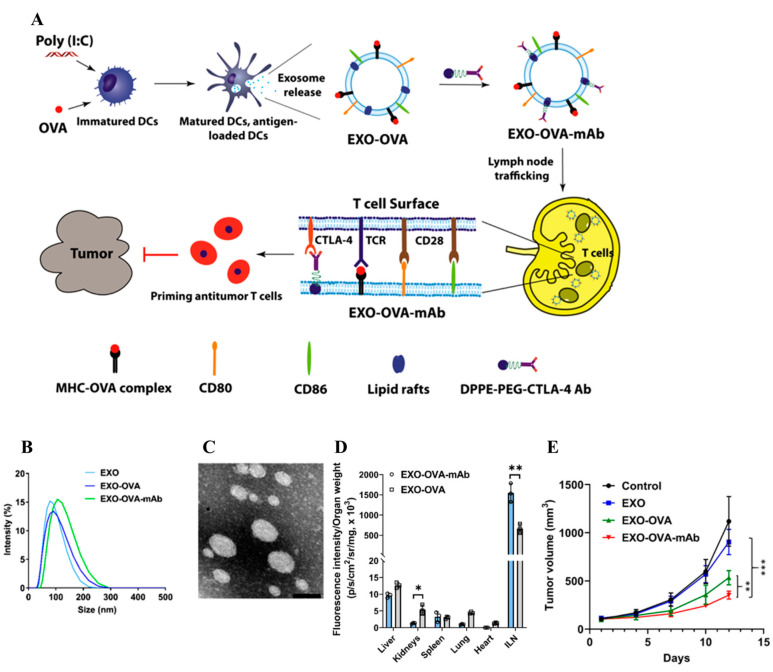
Schematic illustration of the preparation, characterization, lymphatic drainage, and therapeutic effects of activated DC-derived exosomes. (**A**) Activated DC-derived exosomes were prepared using poly(I:C) and OVA and further decorated with aCTLA-4. (**B**) DLS size distribution of exosomes. (**C**) TEM images of exosomes. (**D**) Quantification of the LN accumulation of exosomes. (**E**) Tumor growth curves with exosomes. * *p* < 0.05, ** *p* < 0.01, and *** *p* < 0.001 were analyzed by one-way ANOVA. Figures are reproduced from [126] with permission from Elsevier. Copyright 2020 Elsevier.

Numerous studies have exploited the extracellular vesicles (EVs) secreted from various cell types as a drug delivery platform and therapeutic, as EVs contain miRNA, bioactive lipids, and proteins that can control intercellular communications [125]. As EVs are formed through fusion of the cell vacuole with cell membranes, they can contain various membrane proteins on their surface. Accordingly, EVs from matured and activated APCs could elicit LN-homing ability and immuno-stimulatory functions in antitumor immunotherapy [126,127]. Indeed, EVs released from DCs maturated and activated by OVA antigens and poly(I:C) (30–100 nm) were revealed to contain CD80 and MHC, which stimulate the activation (CD69^+^) of CD8^+^ T cells (CD45^+^CD3^+^CD8^+^). The EVs could efficiently drain into LNs, and their efficacy was improved with a CTLA-4 mAb decoration on the membranes by anchoring lipids-PEG-aCTLA-4 (Figure 6) [126]. In particular, the resulting aCTLA-4 decorated EVs enabled long-term antitumor immunotherapy with effector memory T cells (CD45^+^CD3^+^CD8^+^CD44^+^CD62L^-^) [126]. Similarly, EVs purified from M1 macrophages polarized by LPS and IFN interferon-γ (IFN-γ) (50 nm) [127] displayed significant LNs accumulation, induced the release of proinflammatory cytokines (IL-6) in dLNs, and elicited antitumor immunotherapeutic effects when co-treated with antigens.

### 2.4. Advanced NPs for Deeper Access to the LNs

**Figure 7:**
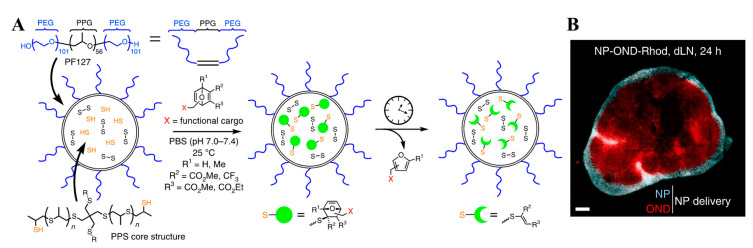
Schematic illustration of the (**A**) preparation of multistage drug delivery systems and (**B**) distribution of cargos in dLNs. Figures are reproduced from [128] with permission from Springer Nature. Copyright 2020 Springer Nature.

After transport to the LNs, immuno-modulatory drugs should access the LN parenchyma where adaptive immune cells reside. However, LN conduits and cortex function as size-dependent exclusion barriers to allow access to solutes that are less than 70 kDa (~several nm in size) [73], which could lead to a size conflict for efficient passive transport [40,41,42,43,44,45,46]. The Susan N. Thomas groups reported a multistage drug delivery system comprising a 30 nm pluronic F127 micelle (30 nm), whose core was stabilized with physically entangled PPS and shell was decorated with a CpG via a linker to exhibit first order degradation independent of pH and solvents (Figure 7) [128]. This size change for the cargo allowed the drug-conjugating NPs to efficiently drain into the LNs via size-dependent passive transport and subsequently deliver drugs to the LN parenchyma [128]. Owing to the efficient delivery of CpG into the LN parenchyma, higher activation (CD40^+^ and/or CD86^+^) of conventional DCs (cDCs, CD45^+^CD11c^+^B220^-^), plasmacytoid DCs (pDCs, CD45^+^CD11c^+^B220^+^), and B cells (CD45+CD11b^-^CD3^-^B220^+^) was observed with NPs with multistage drug delivery compared to that with the controls [128]. Accordingly, higher expansion of T cells (CD45^+^CD3^+^B220^-^), which significantly suppressed EL-4 lymphoma tumor growth in LNs, was recorded [128]. This strategy outlines an approach to re-purpose numerous trojan horse and size-transformable NPs previously reported for deep penetration into the tumor tissues [129,130,131] for LNs-mediated anticancer immunotherapy.

## 3. Advanced HGs for LNs Accumulation and Adaptive Cancer Immunotherapy

**Figure 8:**
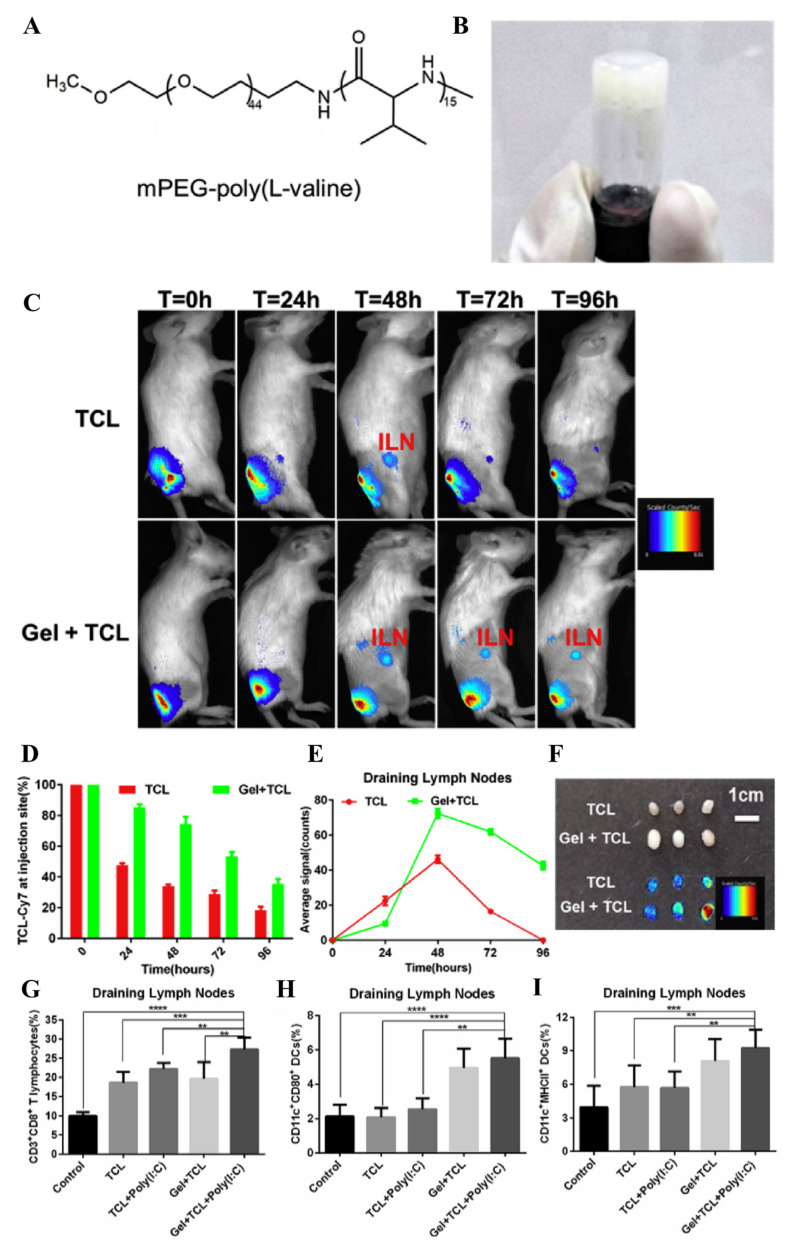
Lymphatic drainage and immune profiles of the tumor cell lysates (TCL) and poly(I:C)-loaded hydrogels. (**A**) Chemical structure of mPEG-poly(L-Valine). (**B**) Image of a hydrogel. (**C**) IVIS images of antigens-Cy7 administered and released from hydrogels. (**D**) Quantification of fluorescence signals at the injection site over time. (**E**) Quantification of the fluorescence signals of antigens-Cy7 at LNs. (**F**) Ex vivo IVIS images of the LNs at 96 h after injection. (**G**–**I**) Populations of (**G**) CD8^+^ T, (**H**) CD80^+^ DCs, and (**I**) MHCII^+^ DCs in the dLNs. ** *p* < 0.01, *** *p* < 0.001, and **** *p* < 0.0001 were analyzed by students’ *t*-test. Figures are reproduced from [171] with permission from Elsevier. Copyright 2018 Elsevier.

According to numerous reports, i.t. administration of HGs facilitates the sustained release of incorporated immunotherapeutics into tumor microenvironments, enabling efficient and/or prolonged anticancer immune responses in tumors [132,133,134,135,136,137,138,139,140,141,142,143,144,145,146,147,148,149,150,151,152,153,154,155,156,157,158,159,160,161,162,163,164] or tumors and TdLNs [165,166,167,168,169]. The immunotherapeutics released from HGs in a sustained manner would also accumulate in the TdLNs. Some studies attempted to determine the effects of sustained release on the lymphatic accumulation of immunotherapeutics, immuno-modulations in LNs, and overall therapeutic effects [31,170,171,172]. An HG prepared via crosslinking between aldehyde-functionalized mannan and *N, O*-carboxymethyl chitosan led to higher accumulations of OVA antigens in dLN at 24 h and 48 h after its locoregional s.c. administration than free OVA and Alum/OVA formulations, which promoted and prolonged the production of antigen-specific immunoglobulin G (IgG), despite a slight delay in tumor growth [170]. In addition, mPEG-*b*-poly(_L_-valine) HGs prolonged and enhanced the lymphatic accumulation of tumor cell lysates that were incorporated in the HGs and released from the injection sites (Figure 8) [171]. Accordingly, significant activation (MHCII^+^, CD86^+^) of DCs (CD45^+^CD11c^+^) in dLNs and expansion of CD8^+^ T cells (CD45^+^CD3^+^CD8^+^) in dLNs, spleen, and tumors were achieved when poly(I:C) and tumor cell lysates were incorporated in the HGs administered s.c. to the tissue i.l. to the tumor [171]. Compared to the control with free formulations, tumor growth could be significantly delayed with HG [171]. Similarly, silk HGs administered s.c. to the tissue i.l. to the tumor facilitated improved accumulation of the immunotherapeutics (herein, neoantigens, CpG, and STING agonist 2, 3-cyclic-GMP-AMP (cGAMP)) in dLNs, leading to significant suppression of tumor growth with the expansion of activated DCs (CD86^+^CD80^+^ CD45^+^CD11c^+^) in dLNs and activated CD8^+^ T cells (41BB^+^ CD45^+^CD3^+^CD8^+^) in tumors [172]. These studies highlight the importance of the pharmacokinetics of immunotherapeutic drugs in LNs, where adaptive immune responses mainly occur. However, the mechanism whereby HG-mediated sustained release improves lymphatic accumulations of cargos has not been clearly revealed.

## 4. Hybrid HGs and NPs for LNs Targeting and Adaptive Cancer Immunotherapy

**Figure 9:**
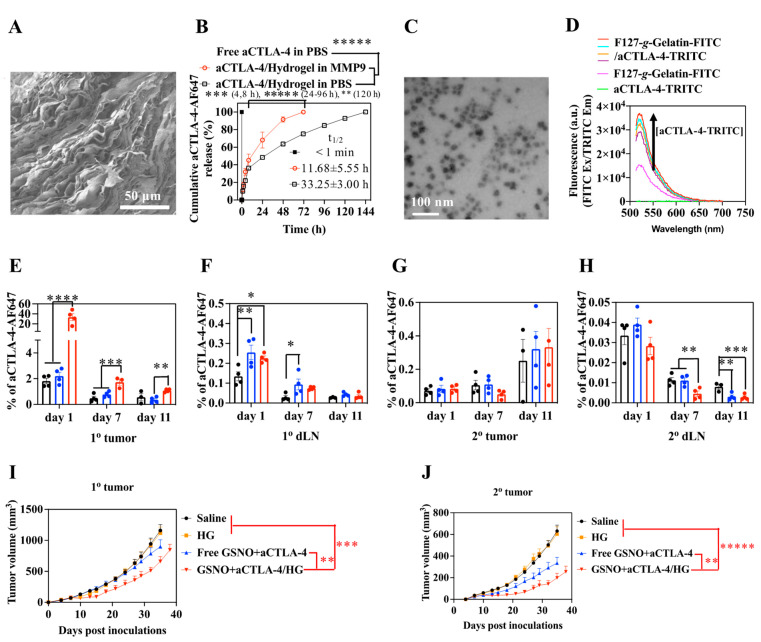
Characterization, lymphatic drainage, and anticancer immunotherapeutic effects of ICB mAbs-loading micelles in situ released from thermosensitive hydrogels. (**A**) Representative SEM image of F127-Gelatin thermosensitive hydrogels. (**B**) Cumulative release of aCTLA-4 mAbs from the hydrogel in response to MMP-9. (**C**) Representative image of the F127-Gelatin micelle in situ released from the hydrogel. (**D**) FRET assay to demonstrate the load of aCTLA-4 mAbs on the in situ micelle via confirmation of the FRET signal between FITC-labeled F127-Gelatin polymer and TRITC-labeled aCTLA-4. (**E**–**H**) Quantification of the fluorescence signals of AF647-labeled aCTLA-4 mAbs released from the hydrogel at LNs and tumor after i.t. injection into the primary tumor (1^o^ tumor). (**I**) 1^o^ and (**J**) 2^o^ tumor growth curves with the hydrogel formulations. * *p* < 0.1, ** *p* < 0.05, *** *p* < 0.01, **** *p* < 0.001, and ***** *p* < 0.0001 were analyzed by one-way ANOVA with Tukey post-hoc for E-H, two-way ANOVA with Tukey post-hoc for B, and ANOVA using linear mixed-effects model for I and J. Figures are reproduced from [174] with permission from Springer Nature. Copyright 2022 Springer Nature.

Hybrid systems have been developed to exploit the potential of HGs and NPs for improved accumulation of immunotherapeutics in LNs. Yin et al. mixed graphene oxide (GO, ~200 nm) with linear PEI (LPEI) to form HGs encapsulating R848 via π−π stacking with GO and OVA-mRNA via electrostatic interactions with LPEI [173]. HG-released NPs (~220 nm) formulated via an electrostatic interaction between R848-loaded GO (negative charges) and OVA-mRNA/LPEI polyplex (positive charges), were released in a sustained manner, and led to markedly higher uptake of cargos into the dLNs when injected s.c. [173]. The prolonged lymphatic delivery of R848 and OVA-mRNA resulted in the most efficient tumor suppression with CTLs (IFN-γ^+^ CD45^+^CD3^+^CD8^+^) compared to that of the controls, including saline, free R848 and OVA-mRNA, and OVA-mRNA-loaded HG [173]. Our group reported a novel polymeric HG to release drug-loaded micelles in situ without using additional NPs (Figure 9) [174]. F127-Gelatin was prepared by crosslinking F127 to gelatin, which increased the hydrophobicity, enabling the formation of a thermosensitive HG at very low concentrations (4–7 wt.%) compared with the precursor F127 (>15 wt.%) [174]. Interestingly, the F127-Gelatin polymers comprising thermosensitive HGs were released as micelles (~30 nm) loaded with aCTLA-4 mAb in a sustained manner [174]. Sustained release and a size appropriate for passive lymphatic transport facilitated the efficient and prolonged accumulation of aCTLA-4 mAb, ultimately enabling efficient anticancer immunotherapy [174]. The concept of NPs and HG hybrid systems is expected to be more extensively explored for efficient locoregional anticancer immunotherapy in the near future.

## 5. Conclusions and Perspectives

From a traditional pharmaceutical perspective, anticancer drugs and their delivery formulations are mainly systemically administered to selectively reach metastatic tumors or deep tumor tissues during blood circulation. Locoregional administration via the i.t. route is occasionally employed to mitigate off-target systemic delivery and improve tumoral accumulations of drugs when tumors are located to be easily accessed to the treatment. 

Advances in biotechnology and expanded knowledge on immunology help highlight lymphatic systems as important targets for adaptive anticancer immunotherapy. However, the systemic administration routes have limitations, such as inefficient lymphatic delivery compared to locoregional delivery [5,31]. Accordingly, in this review, we discussed emerging locoregional drug delivery strategies that target lymphoid tissues instead of tumor and tumor microenvironments for cancer immunotherapy. The physiology of lymphatic systems motivate the use of NPs for the accumulation of immuno-modulatory drugs at LNs, where adaptive immune responses mainly occur, especially via passive lymphatic transport mechanisms. Further, sophisticated engineering of NPs facilitates the exploitation of active lymphatic transport pathways of cargos, which utilize the intrinsic lymph-homing ability of peripheral APCs. In addition to NPs, HGs that release drugs in a sustained manner have demonstrated benefits such as improved, efficient, and prolonged lymphatic accumulation of drugs, avoidance of repeated administrations, and achievement of dose sparing, thereby leading to efficient anticancer immunotherapy. However, the mechanisms associated with sustained locoregional release and lymphatic drainage need to be further addressed. In particular, hybrid NP and HG systems have been explored as a potential platform to combine the benefits of both NPs and HGs. 

There are several issues to be addressed in locoregional anticancer immunotherapy targeting LNs. First, several tumors lack tertiary lymphoid structures [175,176,177,178] where are primary target tissues of locoregional anticancer immunotherapy to instruct and expand effector immune cells. Second, although locoregional delivery allows immune responses specific to the tumors and dLNs and mitigates the systemic immune-related adverse toxicity, it is unclear whether it is beneficial to the treatment of metastatic tumors, compared to systemic delivery to allow the wide distribution of cargos to various LNs presented throughout the body [179,180,181]. Therefore, innovations in the design of NPs and HGs for lymphatic delivery and further investigations to address the issues would maximize therapeutic effectiveness, improve patient compliance, and increase safety in the clinic through locoregional anticancer immunotherapy. 

## Data Availability

Not applicable.
